# New Data concerning the Epidemiology of Hepatitis B Virus Infection in Greece

**DOI:** 10.1155/2008/580341

**Published:** 2008-04-06

**Authors:** Konstantinos D. Pantazis, Ioannis S. Elefsiniotis, Hero Brokalaki

**Affiliations:** Department of Internal Medicine, Faculty of Nursing, University of Athens, 11521 Athens, Greece

## Abstract

There is an obvious, significant, and diachronic reduction of *the prevalence of HBV infection* in Greece, concerning the general population as well as some traditionally high-risk groups, mainly as a result of constant informing and the widespread initiation of preventive and prophylactic measures, as well as the improvement of health care services. Nevertheless, there are special groups and populations (economical refugees, religious minorities, HIV-positive patients, abroad pregnant women, prostitutes, etc.) who represent sacs of high HBV endemicity and need epidemiological supervision and intervention, in order to limit the spread of the infection and to further improve the existing epidemiological data.

Hepatitis B virus (HBV) infection is a major public health problem worldwide, as more than 2 billion people have been infected, whereas more than 350 million present chronic HBV infection. It is estimated that a significant proportion (15–40%) of chronic HBV infected patients develop liver cirrhosis, liver failure, and primary hepatocellular carcinoma (HCC), making chronic hepatitis B one of the 10 major causes of death worldwide [1].

The prevalence of the infection varies among different areas of the world and among special populations within the same area. About 45% of the world population lives in countries in which HBV infection is hyperendemic (South-Eastern Asia and sub-Saharan Africa), where the percentage of hepatitis B surface antigen (HBsAg) seropositivity *outranges* 8% of the total population and the percentage of viral exposure *varies* between 70% and 90%. In these countries, HBV infection is vertically transmitted usually during the perinatal period and horizontally during the infancy and early childhood age, leading to extremely high rates of chronic HBV infection and preserving the reservoir of chronic hepatitis B patients worldwide [2, 3]. Southern countries of Central and Eastern Europe *as well as those of the Mediterranean basin, the Amazon’s sink, Middle East, and Northern Africa* are regions of medium HBV infection seroprevalence (HBsAg seropositivity between 2–7%), whereas countries of North-Western Europe and North America are considered to be countries with low HBV endemicity (HBsAg seropositivity <2%). In these countries, the infection is basically transmitted horizontally during the adolescent and early adult period, mainly by high-risk sexual activity [4].

Moreover, the annual incidence of HCC is extremely high in hyperendemic regions of HBV infection (10–150 cases/100.000 people per year) *compared* to regions of lower prevalence (1–3 cases of HCC/100.000 people per year), a finding that supports the causative relationship of HBV and HCC. The primary HCC is one of the most common tumours (5th in frequency worldwide) and is *considered* responsible for 300 000–500 000 deaths annually, representing the 3rd most common cause of death in the hyperendemic countries of HBV infection [5].

Patients with chronic hepatitis B have a threefold probability to die within a decade compared to general population, mainly by causes directly related to chronic liver disease (HCC, hepatocellular failure, variceal bleeding, etc.) [6]. Regarding the cost of patients’ hospitalization, it seems that patients with chronic HBV infection spend more time in the hospital and need more specialized and expensive in-hospital treatment compared to the general population. An epidemiological and economotechnical study from a set area of Scotland showed a significant difference in the duration of hospitalization and the mean cost of treatment, as well as in the number of admissions and readmissions of patients with chronic HBV infection, compared to the control group [6]. The annual cost of treating patients with chronic HBV infection has been retrospectively estimated and seems to significantly differentiate between patients in procirrhotic or compensated cirrhotic stages of the disease under treatment and those who exhibit decompensated cirrhosis, especially when they undergo liver transplantation procedure. Even the palliative treatment of end-stage HCC costs higher than long-term treatment of early stages of the disease [7].

A significant decline of the incidence of acute hepatitis B cases (67%) has been observed in the USA after 1990, especially among high-risk groups (such as intravenous drug users, homosexual men, and health care professionals), according to Center of Diseases Control (CDC). Widespread vaccination programs, predelivery evaluation of pregnant women and passive-active immunoprophylaxis of infants born from chronic HBV infected mothers, blood donors control, and immunization of the majority of health care professionals, children and teenagers have resulted in that decline. Considering the lack of those preventive and precaution measures in many hyperendemic countries of the world and the massive immigration observed in the last fifteen years, mainly from countries of median or high prevalence of the infection to countries of low HBV prevalence, the epidemiological data of HBV infection *seem* to be significantly modified nowadays.

In Greece, the existing published epidemiological data are difficult to be representative to the general population data, mainly because they are referred to specific populations (blood donors, religious-ethnic minorities, economic immigrants, soldiers, children, pregnant women) or high-risk groups (prisoners, drug users, haemodialysis patients, HIV, prostitutes, etc.) over- or underestimating the problem. The National Institute of Employment and the National Statistical Service reported that the number of recorded foreign immigrants in Greece at the inventory of 2001 was approximately 800 000 (7.2% of recorded Greek population) and the majority of them (65%) *came* from Albania, a country of high endemicity of HBV infection.

In general, the comparison of studies from the early 70s with others from the late 80s shows a significant decline of HBV prevalence in Greece of about 50–80%. Studies on military recruits exhibit a significant decline of HBsAg prevalence, from 4% in 1973 to 0.95% in 1999, mainly due to the modification of socioeconomical and medical practice parameters and to the significant increase of successfully vaccinated people [8, 9]. General population published data are limited in Greece whereas the best-studied group is that of blood donors. In the larger cohort of blood donors studied for 6 consecutive years in Athens, the mean prevalence of HBsAg was 0.84% [10]. A study of a large population of blood donors in Crete island for 5 consecutive years revealed that the mean prevalence of HBsAg (0.4%) was significantly lower than *the one* reported from other compartments of our country [11]. Equally, low percentages of HBsAg prevalence were observed in children of school age and teenagers of a rural population of Crete (0.33%) and in high-risk hospitalized patients (2.66%), reflecting partially the epidemiological data of the general population of the island [12, 13]. A significant decline of HBsAg prevalence by 2.15% and of the percentage of HBV exposure by 22.6% are reported in the first published study concerning the general population, in the region of South-Western Greece, whereas in the region of Epirus in a 3-year prospective study of blood donors, the HBsAg seropositivity *was* of 0.85%, relatively higher than *the one* reported from other compartments of Greece [14, 15].

The immigration of populations from countries of high endemicity of the infection seems to contribute significantly to the rapid modification of epidemiological data, especially in areas bearing the high burden of immigrants. In a serological control of 1020 refugees from South Albania who came and work in Ioannina region, the percentages of HBsAg seropositivity and HBV exposure were 22.2% and 70.6%, respectively. Moreover, a significant proportion of chronic HBV infected Albanian patients were also hepatitis B e antigen-positive [HBeAg(+)] (21.1%) and 12.7% of them were chronic hepatitis D virus (HDV) infected, representing a** population of high infectivity regarding viral hepatitis [16]. Data regarding immigrants living in Athens showed that the prevalence of HBsAg was *significantly* high (15.4%) especially among Albanian and Asian refugees [17]. Likewise, significant differences of the prevalence of HBV infection are reported in the religious minority of Thrace moslems comparing to Greeks from formal Soviet Union who live in the same area and the native habitants of Thrace (9.3% versus 4.3% versus 3.4%, respectively), revealing a population of high endemicity of HBV infection within our country [18].

Perinatal (vertical) transmission is one of the most common ways of HBV infection dispersion, mainly in hyperendemic areas of the world and seropositive pregnant women and their neonates represent the basic target groups for the elimination of vertical transmission, which preserves the main reservoir of chronic HBV infected patients. In the larger group of women at reproductive age studied in Greece, the mean prevalence of HBsAg was 1.15%, but was significantly higher among women of Albanian (5.1%) and Asian origin (4.2%) compared to Greek women (0.29%). The vast majority (71.34%) of HBsAg-positive women were of Albanian origin and about a third of them exhibited significant viral replication at perinatal period [19].

Intravenous drug users and prisoners are also high-risk groups for viral hepatitis who *were studied* epidemiologically in our country. In a study of 544 prisoners who were intravenous drug users concomitantly, 6.5% of them exhibited HBsAg-positivity whereas among prisoners for sexual offences the percentage of HBsAg-positivity *was* higher enough (13%) [20, 21]. The widespread of HBV infection in the Greek drug-users community observed in the past decade, according to published studies [22] seems to be changed nowadays. In an our recently published study, we investigated the presence of serological markers of HBV infection in intravenous drug users (IVDU) with chronic hepatitis C virus (HCV) infection and the results were correlated to the time of drug usage initiation. We found that drug use initiation before 1992 was significantly related to HBV exposure while the vast majority of relatively newer drug users (drug initiation after 1992) were HBV seronegatives [23].

In Greek oncologic patients with solid tumors, the percentages of HBsAg-seropositivity were 5.3% and those of HBV exposure 44%, while a proportion of them (14%) had clinical and/or biochemical exacerbation of liver disease during the chemotherapy schedule; this percentage is expected to be much higher in patients with haematological malignancies, with or without steroid administration according to the international literature [24]. Furthermore, significantly higher prevalence of HBV infection is observed in chronic alcoholic patients—with or without established liver disease—compared to healthy blood donors or nonalcoholic hospitalized patients, which is ascribed to their specific and some times unexpected behavior [25].

Chronic liver disease due to coexisting chronic viral hepatitis B and/or C as well as a consequence of hepatotoxicity of the antiretroviral therapy is one of the most important factors of morbidity and mortality of human immunodeficiency virus- (HIV-) infected patients, since the classic opportunistic infections and the complications of severe immunodeficiency have been significantly diminished. In HIV-seropositive patients the probability of chronic HBV infection and chronic liver disease after exposure to HBV is extremely high and is characterized by very high levels of viraemia (serum HBV-DNA) and extremely low percentages of spontaneous loss and/or seroconversion of HBeAg [26]. About 13% of a group of HIV-positive patients, mainly homosexual men (68.5%), were HBsAg-positive and the percentage of their exposure to HBV infection was 67.4%, representing a high-risk group for viral hepatitis [27]. In a cohort study in Greece, regarding 737 HIV-positive patients, the percentage of HBsAg-seropositivity was 12.1% while the majority of HIV/HBV coinfected patients (60.9%) were also HBeAg-positive and they exhibited extremely high levels of HBV viral load, suggesting the direct uptake of preventive measures for this high-risk group, in order to protect its health and the public health in general [28].

An obvious trend of reduction of the HBV prevalence is observed in other traditionally high-risk groups, such as prostitutes, since the percentage of HBsAg-seropositivity (11%) at the early 80s has been reduced to 3.3% in the late 90s; these data need to be reevaluated because of the high percentage of people trafficking for sexual reasons, mainly from countries of median-high HBV prevalence to our country, who are not reported or sanitary controlled and are thought to represent the most significant cause of HBV transmission** in our days [29, 30]. Data from a study of the Department of Public Order were presented in daily press and according to conservative calculations there are more than 14 000 people who are illegally sexually prostituted in our country.

Finally, the professional exposure in the health care worker field has been studied since the 70s, where the percentages of HBsAg seroprevalence were 2.4% for medical students and 4.6% for nursing staff. In a recent study from Hippokration Hospital of Athens including 400 nursing staff, the percentage of HBsAg-seropositivity was 1.25% and the HBV exposure rate was 16%, but only 61% of that high-risk group were efficiently vaccinated, so there is a significant percent of population unprotected for various but nonaccepted reasons [31].

In conclusion, although there is an obvious, significant, and diachronic reduction *of the prevalence of HBV infection* in Greece, concerning the general population as well as some traditionally high-risk groups, mainly as a result of constant informing and the widespread initiation of preventive and prophylactic measures as well as the improvement of health care services, there are special groups and populations (economical refugees, religious minorities, HIV-positive patients, abroad pregnant women, prostitutes, etc.) who represent sacs of high-HBV endemicity and need epidemiological supervision and intervention, in order to limit the spread of the infection and to further improve the existing epidemiological data.
